# Intradural Solitary Fibrous Tumor of the Lumbar Spine: A Distinctive Case Report

**DOI:** 10.1155/2015/708472

**Published:** 2015-01-14

**Authors:** Recep Basaran, Mustafa Kaksi, Mustafa Onoz, Ece Balkuv, Aydin Sav

**Affiliations:** ^1^Neurosurgery Department, Dr. Lutfi Kirdar Kartal Training and Research Hospital, 34890 Istanbul, Turkey; ^2^Neurosurgery Department, Eyub State Hospital, 34050 Istanbul, Turkey; ^3^Neurosurgery Department, Medipol University School of Medicine, 34200 Istanbul, Turkey; ^4^Neurology Department, Istanbul Medeniyet University Goztepe Education and Research Hospital, 34730 Istanbul, Turkey; ^5^Pathology Department, Acibadem University School of Medicine, 34718 Istanbul, Turkey

## Abstract

*Background*. Solitary fibrous tumors are ubiquitous mesenchymal neoplasms of putative fibroblastic origin. They were originally described in the pleura but subsequently have been reported in many extraserosal sites. Solitary fibrous tumors may also occur in the meninges, central nervous system parenchyma, and spinal cord. *Case*. A 67-year-old male patient with progressive lower extremity weakness, urinary urgency, and sexual dysfunction has been admitted to our hospital. On his lumbar MRI, we detected an intradural lesion posterior to the L3 vertebral corpus. We resected the lesion by L3 total laminectomy. Immunohistological findings revealed strong and diffuse immunopositivity with vimentin, CD34, and bcl-2. Ki-67 proliferation index was 5–8%. We did not detect any recurrence 12 months after his operation. *Conclusion*. SFT is mostly seen in young and middle-aged patients and should be considered among differential diagnosis in cases suffering from pain, hypoesthesia, and urinary dysfunction. Gross total resection should be primary treatment. Tumors that have high Ki-67 labeling should be followed up for potential recurrences.

## 1. Introduction

Solitary fibrous tumors (SFTs) are ubiquitous mesenchymal neoplasms of putative fibroblastic origin [1]. They were originally described in the pleura and first described by Klemperer and Rabin in 1931 but subsequently have been reported in many extraserosal sites [2, 3]. Solitary fibrous tumors may also occur in the meninges and central nervous system parenchyma [4]; in addition, spinal cord and spinal nerve involvement has been reported [4–6].

We describe a rare SFT case of the lumbar spine which presented with a few minor neurological deficits. We also discussed its surgery and histopathologic and immunohistochemical features that will likely be of utmost importance in the follow-up of the patient.

## 2. Case Report

**Figure 1:**
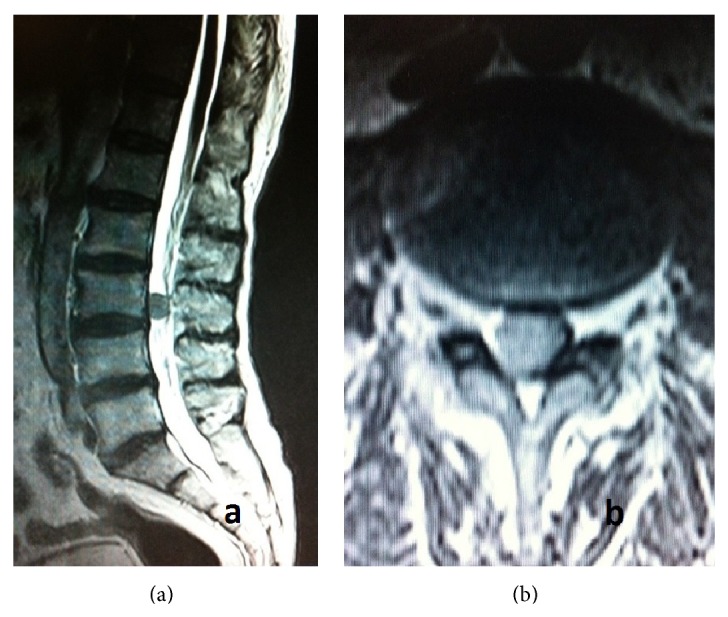
(a) Preoperative sagittal T2-weighted lumbar MRI demonstrated well-circumflanced, hypointense lesion anterior to L3 vertebrae. (b) Preoperative axial T2-weighted lumbar MRI demonstrated the well-circumferenced lesion with slightly hyperintense signal according to neural structures and hypointense signal according to CSF.

A 67-year-old male patient was admitted to our department with progressive walking disability for the last 6 months. Moreover, the patient had experienced urinary urgency and sexual dysfunction for the last 3 months. His physical examination was within the normal limits. Neurological examination revealed paresis of bilateral iliopsoas and rectus femoris muscles. The iliopsoas muscle showed a 3/5 motor loss on the right side and 4/5 on the left side. Additionally bilateral rectus femoris muscle deficit was 4/5. The patient had hypoesthesia in areas that corresponded to the L3 and L4 dermatomes. His lumbar T2-weighted magnetic resonance imaging (MRI) showed a well-circumfluenced and hypointense lesion with 1.3 × 1.5 × 1.5 cm that was located in posterior of the L3 vertebral corpus. This lesion was pushing intradural neural structures to the left side and anterolaterally (Figures 1(a) and 1(b)). The lesion was well-circumfluenced with homogenous contrast enhancement. Cranial, cervical, and thoracic MRI revealed no other pathology.

### 2.1. Surgery

A L3 total laminectomy was performed on the patient. Upon the 2 cm vertical incision of bulky dura, it was bulky and, during the exploration of dural sac, highly vascularized and well contoured lesion was pushing rootlets anteriorly. The lesion was brown discolored without invading dura and minimally adhesive. There was no evidence of mass effect or invasion over the root. Initially this particular lesion was released from dura and then from the adjacent arteries; venous connections of the lesion were completely resected. Clinical and neurological symptoms were related to cauda equina syndrome that was secondary to the mass compression. His postoperative neurological examination showed bilateral ameliorated muscle strength which was accompanied with 4/5 of iliopsoas and rectus femoris muscles.

### 2.2. Histopathology

All formalin-fixed, paraffin-embedded (FFPE) surgical materials were sectioned and stained routinely with hematoxylin and eosin; selected sections from surgical material were stained with Gomori reticulin and Masson's trichrome stain. Immunohistochemical stains for epithelial membrane antigen (EMA, Neomarkers [E29]), BCL-2 (SYCTEK [12  4]), vimentin (SYCTEK [v9]), S-100 (SYCTEK [4c4.9]), progesterone (NOVOCASTRA [PGR-312]), smooth muscle actin (SMA) (SYCTEK [sm-1]), desmin (SYCTEK [DE-R-11]), CD34 (SYCTEK [QBend/10]), p53 (SYCTEK [DO/7]), and Ki-67 (DAKO [MIB-1]) were performed on selected sections with appropriate positive and negative controls.

**Figure 2:**
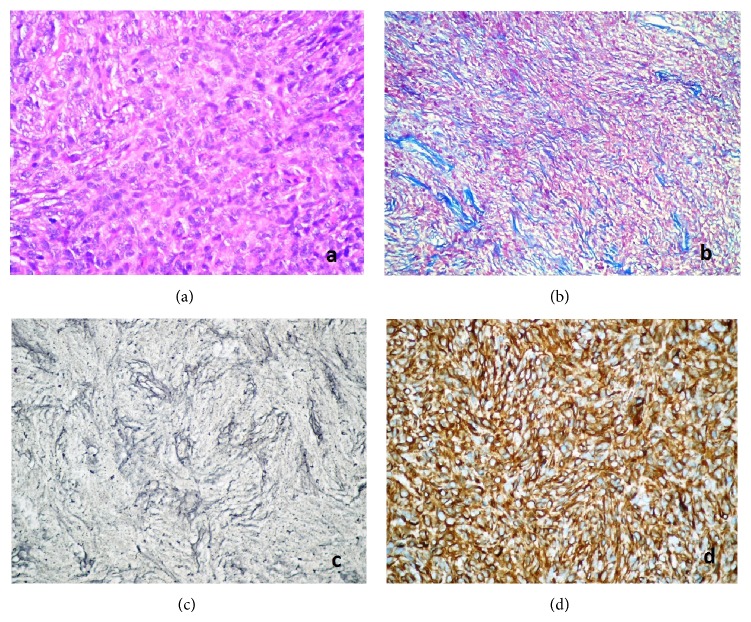
(a) Uniform spindle cells arranged in interlacing fascicles with deposition of scanty collagen among tumor cells (HE ×400). (b) Scarce collagen network was demonstrated among tumor cells with Masson's trichrome stain (MTC, ×200). (c) Scarce reticulin network was demonstrated between many tumor cells with Gomori reticulin (Gomori's reticulin, ×400). (d) Strong and diffuse immunopositivity with vimentin (streptavidin biotinylated complement; vimentin ×400).

Histological examination revealed uniform spindle cells arranged in interlacing fascicles with deposition of scanty collagen between cells (Figure 2(a)). Tumor cells were with elongated nuclei with slightly granular chromatin. Minute areas showed extensive fibrosis and hyalinization. Focal areas of staghorn like vascular channels imitating those of hemangiopericytoma were spotted. No evidence of mitotic figures was present. Pleomorphism and necrosis were absent. No psammoma bodies and whorls were identified. Therefore, the histology parameters supported a benign spindle cell tumor. Masson's trichrome stain showed scarce collagen network between many tumor cells (Figure 2(b)). Scarce reticulin network was demonstrated between many tumor cells with Gomori reticulin (Figure 2(c)).

**Figure 3:**
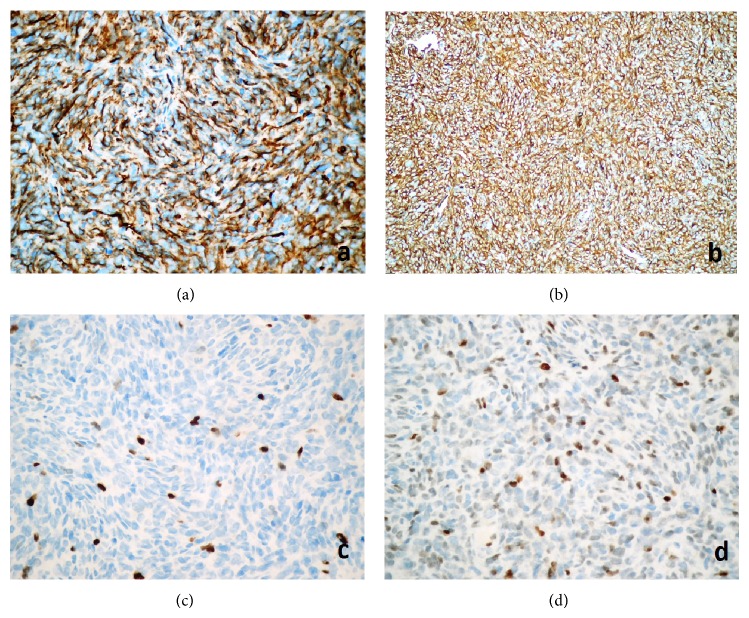
(a) Strong and diffuse immunopositivity with CD34 (streptavidin biotinylated complement; CD34, ×400). (b) Diffuse immunopositivity with bcl-2 (streptavidin biotinylated complement; bcl-2, ×400). (c) Ki-67 proliferation index was 5–8% (streptavidin biotinylated complement; MIB-1, ×400). (d) Nuclear p53 oncoprotein was dispersing but mild in intensity (streptavidin biotinylated complement; p53 ×400).

Immunohistological findings revealed strong and diffuse immunopositivity with vimentin (Figure 2(d)), CD34, and bcl-2 (Figures 3(a) and 3(b)). The tumor cells did not exhibit positive staining for EMA, S-100, smooth muscle actin, desmin, and progesterone. Ki-67 proliferation index was 5–8% (Figure 3(c)) and nuclear p53 oncoprotein was randomly distributed but was mild in intensity (Figure 3(d)).

### 2.3. Follow-Up

The most recent neurological examination of the patient was performed at 12 months postoperationally. His muscle strength was 5/5. His control lumbar MRI showed no evidence of recurrence.

## 3. Discussion

SFT was defined for the first time in 1931 by Klemperer and Rabin as a tumor that originates from pleura [1, 7]. When we look at the literature we see that SFT has been insistently found mostly in pleura but it has also been reported in central nervous system. At a recently published review article (in 2011) by Fargen et al., it is stated that only 30% of central nervous system cases are found on spinal cord [6].

Our case differs from other spinal SFT cases by being located in lumbar area. Even though intradural localization is common on spinal SFT cases, only 37% of these cases are found intradurally and extramedullary [6]. This ratio is very low at lumbar area. On PubMed, there is not a single case of intradural and extramedullary SFT on lumbar area. All reported cases of lumbar SFTs are in extradural locations [8, 9].

When we examined the initial symptoms at admission we appreciated that the majority of symptoms were pain, hypoesthesia, paresis, urinary dysfunction, or combination of these [6]. Similarly, as in our case, the symptoms consisted of paraparesis and loss of sense in lower extremities. Additionally, age and gender of our patient was also similar with patients seen in the relevant literature. 56% of SFT patients are male and most commonly it is seen on patients between 40 and 60 years old. It is also common between 30–40- and 60–70-year-old patients [6].

MRI findings of SFT are usually similar to one another in literature. Fargen et al. reported that two-thirds of lesions were isointense on T1-weighted imaging with the remainder being either heterogeneous or hypointense. Nearly two-thirds of cases were hypointense on T2-weighted imaging with hyperintense being the next most common (17%). Over three-quarters of cases demonstrated diffuse or homogeneous contrast enhancement with gadolinium administration (78%); however, a significant portion demonstrated only partial or heterogeneous enhancement (21%) [6]. SFTs have a good blood supply with a prominent admixture of hemangiopericytic blood vessels, which explains why all the tumors have exhibited gadolinium enhancement on MRI. Present case shows similar features to cases in the literature.

SFTs are just one of the vimentin/CD34/Bcl-2 families of tumors [10]. SFT can be indistinguishable from other meningeal tumors in imaging studies. Histologically they can represent similar features particularly on hematoxylin and eosin staining, with other spindle cell neoplasms such as schwannoma, fibroblastic meningioma, or hemangiopericytoma [11]. Typically, histopathological features of SFTs are spindle cells embedded in a fibrous matrix in a patternless architecture and alternating hypercellular and hypocellular areas with perivascular hyalinization or myxoid degeneration. A hemangiopericytoma-like vascular pattern is usually present. SFT might show staghorn-type branching of vessels accompanied by bland cell morphology.

Immunohistochemically, they are strongly stained with CD34 and vimentin but are unlikely stained with EMA or S-100 protein [4, 12].

Solitary fibrous tumors arising in spinal nerve roots must be differentiated from schwannoma, meningioma, and hemangiopericytoma. Albeit morphologic features may be helpful. Schwannoma often shows alternating Antoni A and Antoni B areas. Meningioma frequently reveals cellular whorls and psammoma bodies. Finally differential diagnosis depends on immunohistochemical features of this tumors. Schwannomas are invariably S-100 protein positive and meningiomas are epithelial membrane antigen (EMA) positive, whereas both antibodies are absent in SFT. About half of hemangiopericytomas are CD34 immunoreactive, at least focally. They also share many histologic features with SFTs, except for dense collagen bands, which are generally absent in hemangiopericytomas. They probably belong to the same tumor spectrum, and the same features (necrosis, cellular atypia, and mitotic activity) are predictive of malignancy in both hemangiopericytomas and SFTs. According to some authors, distinguishing between questionable cases may not be important.

In our case, histopathologically, there were extensive fibrosis and hyalinization, staghorn like vascular channels. However, there was no evidence of mitotic figures or necrosis or psammoma bodies. Immunohistological findings revealed strong and diffuse immunopositivity with vimentin, CD34, and bcl-2. The tumor cells did not exhibit positive staining for EMA, S-100, smooth muscle actin, desmin, and progesterone. Desmin and S-100 immunoreactivity were also rare in the literature such as in our case [6]. Liu et al. suggest that there was no prognostic significance to progesterone receptor (PR) biomarker expression [13]. However, another study claimed that low expression of progesterone receptors was associated with a worse outcome (*P* < 0.05) [14].

## 4. Conclusion

Spinal lumbar intradural extramedullary solitary fibrous tumor is very exceptional and should be considered among the differential diagnoses of spindle cell tumors. Immunohistochemistry is crucial for the accurate diagnosis of SFT.
